# Comment on Rinaldi et al. Assessment of Response and Safety of Bulevirtide Treatment in Patients with Chronic Delta Virus Infection: The ARISTOTLE Pilot Observational Study. *Viruses* 2025, *17*, 251

**DOI:** 10.3390/v17091227

**Published:** 2025-09-08

**Authors:** Muhammad Hassan Saeed, Muhammad Ahmed Zaheer, Bhunesh Kumar, Arslan Ali, Muhammad Ahmed Shaikh

**Affiliations:** 1 Jinnah Postgraduate Medical Center (JPMC), Karachi 75510, Pakistan; mashaikh18239@gmail.com; 2 Sindh Medical College, Jinnah Sindh Medical University (JSMU), Karachi 75510, Pakistan; muhammadahmedzaheer927@gmail.com (M.A.Z.); bhuneshkumar327@gmail.com (B.K.); ashraf3arslan@gmail.com (A.A.)

Hepatitis D virus (HDV) infection poses a serious global health burden due to its rapid progression to cirrhosis, hepatic decompensation, and hepatocellular carcinoma. Despite being a defective satellite virus, HDV remains one of the most aggressive forms of viral hepatitis, relying on hepatitis B surface antigen (HBsAg) and specifically utilizing the sodium taurocholate co-transporting polypeptide (NTCP) for hepatocyte entry [1]. Pegylated interferon-alpha has been the mainstay, though its limited efficacy and considerable adverse effects have highlighted the urgent need for targeted therapies. Bulevirtide (BLV), an entry inhibitor that competitively blocks NTCP, represents a promising therapeutic advancement.

In this context, we read with great interest the recent article by Rinaldi et al., entitled “Assessment of Response and Safety of Bulevirtide Treatment in Patients with Chronic Delta Virus Infection: The ARISTOTLE Pilot Observational Study” [1] This real-world study offers valuable insights into the use of bulevirtide (BLV) in chronic hepatitis D virus (HDV) infection and provides encouraging data on its short-term safety and efficacy in an Italian cohort [2]. While the authors should be commended for their contribution, several methodological limitations demand clarification to ensure appropriate clinical translation and to guide future research.

A major concern is the simultaneous use of nucleos(t)ide analogues such as tenofovir or entecavir in most patients. This co-administration presents a confounding factor that obscures the ability to isolate the antiviral effects of BLV monotherapy [3]. We acknowledge the authors’ clarification that NUC therapy was administered in accordance with current guidelines and was necessary in a cirrhotic population. Nonetheless, this reinforces our original concern: that the real-world design, while valid, limits the ability to discern BLV’s independent efficacy and safety. Although studies suggest NUCs do not affect HDV RNA suppression directly, their concurrent use without stratified subgroup analysis (e.g., BLV + NUC vs. NUC-only) hinders precise attribution of both efficacy and tolerability [4]. Without a monotherapy comparator arm or clear analytic separation, both the antiviral effect and safety profile of BLV remain difficult to interpret with confidence.

Secondly, while the authors reference the use of vitamin D, they do not provide critical data such as the number of patients receiving supplementation, dosing details, or any statistical analysis to support a potential association with treatment outcomes [5]. We appreciate the authors’ acknowledgment that vitamin D data were not systematically collected and thus precluded formal analysis. However, since its use was referenced in the original article, the absence of structured data collection, dosing information, and outcome correlation renders any implied association with treatment response speculative. Future studies should either report such variables with analyzable data or clearly label them as exploratory to avoid interpretive bias.

We also acknowledge the authors’ rationale for using transient elastography, particularly given the procedural risks in patients with portal hypertension. While non-invasive methods are practical and widely accepted, liver biopsy remains the standard [6]. Future studies may benefit from complementing elastography with standardized scoring systems to improve fibrosis staging clarity in real-world cohorts.

Lastly, while we acknowledge that data were collected from 13 tertiary hepatology centers across Italy, the relatively small sample size and absence of predefined treatment stratification still limit the statistical power and generalizability of the study. We appreciate the authors’ efforts to address this through an ongoing expanded phase involving a larger multicenter cohort with longer follow-up. To strengthen future investigations, we continue to recommend employing well-categorized cohorts with defined treatment arms, transparent reporting of concomitant therapies, and, where feasible, histological confirmation of liver fibrosis. These enhancements will improve the reliability of efficacy and safety outcomes attributed to bulevirtide in chronic HDV infection.
